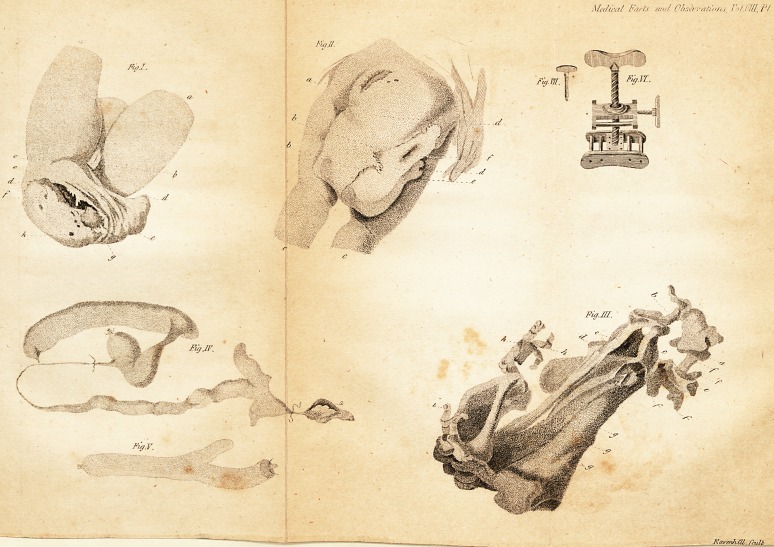# A Case of Monstrosity in a Child; with Physiological Remarks

**Published:** 1800

**Authors:** W. Simmons

**Affiliations:** Member of the Corporation of Surgeons of London; and Senior Surgeon to the Manchester Infirmary.


### MEDICAL FACTS

AND

### OBSERVATIONS.

I. *A Case of Monstrosity in a Child; with physiological Remarks. By Mr.* W. Simmons, *Member of the Corporation of Surgeons of London; and Senior Surgeon to the Manchester Infirmary.*

EXPERIENCE hath sanctioned the propriety of recording such deviations from the usual structure of the body as are discovered by dissection; for, though insulated facts may add nothing to the stock of our knowledge, a collection of cases of a similar character, may illustrate an obscure or mistaken law of the animal economy, and tend to important improvements in the treatment of diseases. Sometimes an observer is so fortunate as to meet with a case, which clearly evinces the impropriety of an opinion formerly entertained; and, ſhould the following be of this deſcription, no further apology will, I truſt, be neceſſary for laying it before the public.

Martha Nuttall, the mother of this child, is a ſtout healthy looking woman, 35 years of age, and has always enjoyed a good ſtate of health. Her huſband alſo is a ſtout, healthy looking man, and their four former children, two boys and two girls, are healthy and free from any perſonal defect. This, their fifth child, was born on the 2d of October, 1793, after a natural labour. Immediately after birth a large tumour was diſcovered, attached to the lower part of the ſpine, which, at the age of nine months, induced the parents to bring her to the Infirmary, where ſhe was admitted under my care.

On a curſory examination, the monſtroſity had the appearance of a child's body, from the pelvis downward; but ſo diſtorted as to render it difficult to give appropriate names to its ſeveral parts. In colour, and to the touch, it correſponded to the perfect body. The bony portion reſembled the bones of the pelvis, widely ſeparated from each other at the ſymphyſis phyſis pubis, and two diſtorted feet annexed. On an anterior view, a large cavity preſented, into which her urine uſually dribbled; the motion of the tumour backward being ſo confined, as not to admit of a ſufficient retraction to prevent its lodgment. A membranous fold had been ſuſpended from the upper edge of this cavity, that, at the time of birth, reached to the hinder part; it had formed a ſort of covering to the cavity, but had been waſted by continued friction to the breadth of about two inches.

On the upper and back part inclining to the left ſide, was a rugoſe appearance, that reſembled an anus imperforated.

The monſtroſity had no paſſage through it.

In other reſpects ſhe was a fine, lively child; and by the aſſiduous attention of her mother ſhe had been kept clean, and free from any excoriation that might otherwiſe have been occaſioned by the lodgment of the urine.

The parents had brought her to the Infirmary for the purpoſe of having the monſtroſity removed by a ſurgical operation, ſhould it be deemed practicable. But this, though the only mode of relief, had an aſpect ſo truly formidable, that it required much previous confideration. And, as from her age fhe was expofed to the hazards ufually attendant on dentition, and on the common eruptive difeafes of infancy, none of which fhe had undergone, it was determined, in confultation, to wait for a more favourable period. She was immediately inoculated for the fmall-pox, and had a mild difeafe.

Until three months prior to her death, her growth had not been at all impeded, and the monftrofity had alfo increafed proportionally. About this time, a fwelling appeared near to the rugofe part above defcribed, which put on the appearance of a phlegmon, but had a peculiarly elaftic feel. When increafed to the fize of an orange it began to ulcerate; her general health then fuffered materially; and the ulcer difcharged an ichorous fluid in confiderable quantity, attended with fymptoms of irritation.

She funk under this train of fymptoms on the 16th of September 1795, when nearly two years of age.

During her indifpofition, the medical courfe confifted of foothing and anodyne remedies and applications; and a generous diet had been been directed, to enable her better to support the increasing discharge from the ulcer.

After death, the parents consented to have the body inspected; and permitted me to take the monstrosity for a preparation.

As it had been thought justifiable to attempt the extirpation of it, had the child lived to a proper age, I was desirous to know how far it would have been practicable; and, if the child could have survived the operation, what chance there would have been for her recovery. Accordingly, I formed a flap as I had intended had the operation been performed during life, and then dissected down to discover the principal attachment between the two bodies. It then appeared that the union consisted merely of a ligamentous substance, which connected the monstrosity to the point of the os coccygis of the child, and might have been readily divided; but this could not be known till after death, nor the number nor size of the connecting blood-vessels. Only one artery was discovered, about the size of a crow-quill; and one nerve of nearly equal diameter.

Had the child lived to undergo the operation, it is probable, therefore, that she would have recovered.

Nothing unufual appeared on infpecting the cavity of the thorax and abdomen, excepting in the latter a *cul de fac* that proceeded from the inteftine ileum. Nor could any communication be traced between either of thefe cavities and the monftrofity. The fwellings in the groins had been occafioned by the morbid ftate of the inguinal glands.

Befides the parts above defcribed, the monftrofity confifted of a mafs of fat, that contained in its centre a clofed inteftine, curioufly fupended over a projection of bone, and fecured from difplacement by its proper ligament.

In the plate of the fkeleton, a ligament will be obferved, that is fituated on each fide of an excavation in the bone, that had fome refemblance to the hollow of the facrum; it contained a medullary fubftance in the recent ftate, and the ligaments feemed to correfpond to the facro-fciatic.

The clofed inteftine meafured more than a foot in length; was of an equal diameter, as will be feen by the plate, and at the opened end of a ftronger texture: but it confifted of one cavity only, which contained a fluid fimilar in colour and confiftency to the meconium found in the bowels of a new-born child.

I believe that no inſtance of monſtroſity ſimilar to this is to be found on record. Baron Haller in his elaborate work " De Monſtris," publiſhed in the third volume of his " Opuſcula minora," has made a large collection of facts, but he has not recorded any caſe in which a cloſed inteſtine was diſcovered that contained meconium, as in the preſent inſtance: on the contrary, he ſays expreſsly, " Inteſtina in omnibus paulo prius, aut ſerius conjuncta." * Perhaps the *cul de ſac* had been an abortive attempt to connect the inteſtines of the child with the cloſed inteſtine of the monſtroſity.

As it had been an opinion generally entertained, that the fœtus in utero received nouriſhment by the mouth, it neceſſarily followed, that the contents of the inteſtines, at the time of birth, ſhould be conſidered as the refuſe of aliment unaſſimilated by digeſtion. But, in this caſe, the cloſed inteſtine of the monſtroſity could have no communication with the inteſtines of the child; and we muſt reſort to ſome other mode of accounting for the production of the fluid contained in it.

* De Monſtris, lib. 2. pag. 164.

The growth of the body after birth, depends on the digeſtion of food taken into the ſtomach, which is firſt converted into chyle, and then into blood. In the fœtus, however, the paſſage to the ſtomach has been ſometimes wanting, and yet it had attained to a conſiderable size.* It is, I believe, well aſcertained, that blood is formed in the embryo, ſoon after conception, and prior to the exiſtence of the ſtomach; and if the action of its veſſels could produce this fluid in that early ſtate of exiſtence, we may be allowed to ſuppoſe that the matter had been furnished by the mother?

The proceſs employed by nature in augmenting the body after birth, is chiefly by the flow of arterial blood; and it appears that this may be ſupplied to the fœtus in utero, by a more simple process than that of digeſtion. We know that in the uterine ſtate, the placenta ſupplies the office of lungs, which are then wholly paſſive; and it does not appear to be

* Dr. Monro, the preſent celebrated, profeſſor of anatomy, has publiſhed an inſtance of this kind of monſtroſity in the third volume of the Tranſactions of the Royal Society of Edinburgh.

at all more neceſſary for perfecting the fœtus, that the ſtomach ſhould be excited into action.

An objection to this opinion ſeems to ariſe from the funis umbilicalis having been found obliterated, even when the fœtus had attained to a conſiderable ſize; but I think it probable that connecting veſſels, of a very ſmall diameter, may be ſufficient for carrying on the neceſſary degree of circulation. And when the fœtus had been expelled free from marks of putreſcence, the obliteration had probably taken place a ſhort time only before its excluſion, as it had been placed under circumſtances moſt favourable to putrefaction. The neceſſity of air being thus conveyed to the fœtus, ſeems to ſtrengthen this conjecture.

If the meconium be fæces, its colour might be preſumed to depend on the preſence of bile; and to aſcertain this point, I inſtituted the following experiments:

### Experiment I.

I added about ten drams of diſtilled water to the firſt evacuation of a child after birth, and by repeated agitation, the whole formed a turbid mixture, which, after ſtanding ſome hours, hours, precipitated a mucous fediment to the bottom of the glafs, leaving the fupernatant fluid of an olive green.

### Experiment II.

To three drams of this fluid, carefully decanted, I added twelve drops of the vitriolic acid; it foon became more tranfparent, with a flight change of colour, approaching to an olive green, which, however, had difappeared next day.

### Experiment III.

To three drams of the fluid in another glafs, I added a fmall quantity of ftrong vinegar, but it produced no perceptible change.

Could the prefence of bile be afcertained by an admixture with acids, it is reafonable to infer that none here exifted; and a mixture of bile and water turns immediately green on an acid being added. Had the deep colour of meconinm been owing to bile, it muft have been contained in a quantity fufficient to have produced a dark green, yet fcarcely any change was perceptible.

The

The above, and other experiments, have ſatisfied me, that bile is not contained in the meconium. Indeed, it was impoſſible that bile ſhould form a conſtituent part of the meconium contained in the blind inteſtine of this monſtroſity.

We find from bile, urine, ſynovia, &c. being found, on inſpecting the body of a ſtill-born fœtus, that the glands perform their ſeveral functions, before birth; and as the ſtate of exiſtence before and after birth is ſo materially different, we might be led to conclude, that the ſecretions were intended for a different purpoſe; the urine, for example, as an excrementitious fluid, could anſwer no ſalutary purpoſe in the ſyſtem before birth. It is a fact, well aſcertained, that by depriving an inveſting membrane of its moiſture, its ſides will adhere; and therefore, had no ſecretion taken place, the cavities muſt have been obliterated. It is particularly neceſſary that the hollow viſcera ſhould be in a ſtate fit for immediate uſe, after birth; and accordingly, not only the inveſting membranes are moiſtened by their reſpective ſecretions, but the receptacles of the bile and urine are alſo diſtended with their reſpective fluids. Now, as I do not

ſee

ſee any neceſſity for digeſtion to take place in the uterine ſtate; and, from the facts and arguments above adduced, thinking it at leaſt doubtful; I have been led to conſider the meconium as a ſecretion, from the villous coat of the inteſtines, formed for the purpoſe of preventing the union of their ſides; and in this way I ſhould be diſpoſed to account for the production of the meconium, contained in the cloſed inteſtine of this monſtroſity.

Theſe conſiderations have led me to believe, that the ſecreted fluids formed before birth, are deſtined to prevent the *coaleſcence of the ſides of cavities, and to facilitate motion.*

It would appear that this had been originally intended for a twin-caſe, and that by ſome accident ſoon after conception, a part of one ovum had been deſtroyed.

I feel great diffidence in touching on the ſubject of generation; but truſt that I may be permitted to ſtate a few points, illuſtrative of ſome opinions contained in this paper.

Dr. Harvey's doctrine that all animals proceed "ex ovo", admits, I believe, of ſome exceptions; but the ſubject is not rendered leſs obſcure by the ſuppoſition of an organic filament ment,* which may form itſelf into a perfect being by ſucceſſive acts of volition. In conducting our enquiries, we ſhould, at leaſt, be governed by known facts ; and, as the will has no power over the organic ſtructure of the body after birth, it would be difficult to conceive, how it ſhould produce ſo aſtoniſhing an effect in utero. Beſides, the nerves have been generally admitted to be the medium of volition ; and yet the growth of the fœtus had not been materially impeded, even when no nervous ſyſtem exiſted.†

It appears from experiments, that after a fruitful intercourſe between the ſexes, an ovum is extricated from either ovarium, that it is tranſmitted into the womb by the fallopian tube, and that it then becomes attached to the inner ſurface of the uterus. This ovum or veſicle is at firſt tranſparent, but ſoon becomes organized, and the ſeveral parts are then ſeen

* See Zoonomia, chap. on generation, vol. I.

† A caſe of monſtroſity has been publiſhed by Dr. Clarke, in which no nerves could be traced on diſſection; yet the complicated maſs had attained to a conſiderable bulk. See Philoſophical Tranſactions for 1793, part II. page 154, and Medical Facts and Obſervations, vol. VII. pag. 109.

ſhooting forth in ſucceſſion. Thus far, I believe, is certain; but whether the rudiments of the fœtus are contained in the ovum, or what ſhare is allotted to the male, is not at all known.* The change in the ovarium, after the extrication of an ovum, is demonſtrable; and is known by the name of *corpus luteum.*

The texture of the ovum, ſoon after conception, muſt be ſo delicate, that any violence acting upon it, would probably deſtroy the whole, and produce an abortion; but when a ſmaller force had been applied, only a part might be deſtroyed, or its arrangement diſordered. Should two ova become impregnated, I ſee no difficulty in conceiving, that the partial deſtruction of one might produce its union to the other. In this way I ſhould be diſpoſed to account for the production of this monſtroſity, for when a part of one ovum had been deſtroyed, the ſame violence might derange the order of the other, which coming into contact with the perfect ovum, an union

* Dr. Haighton has publiſhed an ingenious paper on generation in the Philoſophical Tranſactions, for 1797. I wiſh the Doctor had aſcertained, when conducting his experiments on rabbits, whether the ſexual diſtinction had been at all influenced by the deſcent of the ovum from the right or left ovarium.

of of their ſurfaces would take place. The property of the living body to form an inoſculation of veſſels, between ſurfaces lying in contact, is beautifully illuſtrated by Mr. Hunter's well known experiment of tranſplanting a human tooth into the comb of a cock.

On the ſuppoſition too of the partial deſtruction of an ovum, I ſhould alſo be diſpoſed to account for the exiſtence of teeth, hair, and other bodies, which have been ſometimes found in the ovaria, in the virgin ſtate.

I wiſh it had fallen to the lot of one better qualified to give an account of this ſingular caſe of monſtroſity; it appears to me to prove deciſively, that meconium is not the fæces of the child in utero. As this fact opened a wide field for conjecture, I have indulged in a few ſhort excurſions, at the hazard of reproof: but I muſt here deprecate the ſeverity of criticiſm, and truſt that the above hints, and vague conjectures, will be received with candour, and commented upon with indulgence.

### Explanation of the Figures,*

(Plate I.)

Fig. I. *Anterior view.*

*aa* Thighs turned upwards.

*b* Pudendum.

*c* Attachment to the perfect body.

*dd* Outer angles of the monstrosity, which, to the touch during life, resembled the ossa pubis separated at the symphisis.

*ee* The ragged edge of the membranous fold.

*f* An imperfect hand.

*g* An imperfect foot.

*h* Cavity into which the urine used to dribble.

Fig. II. *Posterior view.*

*a* The rugose appearance resembling an imperforated anus.

*bb* The ragged edge of the membranous fold.

*cc* Thighs of the perfect body.

*dd* The monstrosity.

* The drawings from which these figures have been engraved, are of two thirds of the natural size; in the engravings, the scale is reduced to one third of the size of the drawings. Editor.

Fig. I.

Fig. II.

Fig. VII. Fig. VI.

Fig. IV.

Fig. V.

Fig. III.

Ravenhill sculp

*e* An imperfect extremity better ſeen in the anterior view.

*f* A ſlight depreſſion.

Fig. III. *View of the ſkeleton.*

*a* Lower part of the ſpine ending in the os coccygis of the perfect body.

*b* The ligamentous ſubſtance connecting the monſtroſity to the perfect body.

*cc* Ligaments correſponding to the ſacro-ſciatic.

*d* Cavity containing medullary ſubſtance in the recent ſtate.

*e* Bony proceſs over which had been ſuſpended the cloſed gut repreſented in *fig. iv.* —*the dotted line marks the direction of the ligament that confined it, but which* was incautiouſly deſtroyed in the diſſection.

*ffff* Irregular bony proceſſes.

*ggg* Tibial bones.

*hh* Two feet probably, from the ſeparation of the bones in the ſkeleton, which appeared as one in the recent ſtate.

*i* A diſtorted hand or foot.

The other parts have no diſtinct reſemblance to any bones in a perfect ſkeleton.

### Fig. IV.

a Part of the cloſed inteſtine, that contained meconium, the coats of which were ſtronger than the reſt of it. The contracted portion, being punctured accidentally, was ſecured by ligatures for the purpoſe of inflating it with air. The meconium was let out at the end adjoining.

### Fig. V.

Part of the jejunum, in the perfect child; with a proceſs from it terminating in a blind pouch.

## Figures and Tables

**Fig.I. Fig.II. Fig.III. Fig.IV. Fig.V. Fig.VI. Fig.VII. f1:**